# Rare ocular features in a case of Kabuki syndrome (Niikawa-Kuroki syndrome)

**DOI:** 10.1186/1471-2415-14-143

**Published:** 2014-11-24

**Authors:** Yi-Hsing Chen, Ming-Hui Sun, Shao-Hsuan Hsia, Chi-Chun Lai, Wei-Chi Wu

**Affiliations:** Department of Ophthalmology, Chang Gung Memorial Hospital, No. 5, Fu-Hsing Street, Kweishan, Taoyuan, 333 Taiwan; College of Medicine, Chang Gung University, Taoyuan, Taiwan; Department of Pediatrics, Chang Gung Memorial Hospital, Taoyuan, Taiwan

**Keywords:** Kabuki syndrome, Colobomatous microphthalmia, Optic disc dysplasia, MLL2 gene mutation

## Abstract

**Background:**

Kabuki syndrome is a multi-system disorder with peculiar facial features, and ophthalmic abnormalities are frequently involved. This case report of a child with Kabuki syndrome describes two new previously unreported ophthalmic conditions.

**Case presentation:**

A 3-year-old Taiwanese boy with Kabuki syndrome had a short stature, spinal dysraphism, intellectual disability and typical facial features. Ophthalmic findings which have been previously reported in the literature and in this patient, included ptosis, esotropia, coloboma of the iris, retina, choroid and optic disc, and microcornea. The newly identified ophthalmic features in this patient included colobomatous microphthalmos and a dysplastic and elevated disc without central cupping. The genetic analysis identified an MLL2 gene mutation.

**Conclusion:**

The presentations of a dysplastic disc and colobomatous microphthalmia are rarely reported in patients with Kabuki syndrome, but these ophthalmic abnormalities may affect vision. Detailed ophthalmic evaluations in children with Kabuki syndrome are advised.

## Background

Kabuki syndrome is a rare multi-system disorder that was first described in Japan [1, 2]. To date, approximately 350 patients have been identified [3–5]. The principle diagnostic criteria for Kabuki syndrome include a short stature, skeletal anomalies, dermatoglyphic anomalies, intellectual disability, and characteristic facial features that resemble the make-up worn in Japanese Kabuki theatres [6]. However, other features, such as congenital heart disease, cleft palate, deafness and ophthalmic abnormalities, usually in the form of strabismus and ptosis, have been reported [3–5]. Most cases are sporadic, but familial cases of MLL2 gene mutations also exist [6–8]. We describe a case of Kabuki syndrome with an MLL2 gene mutation and rare features of colobomatous microphthalmos and a dysplastic optic disc. We also discuss the clinical overlap with CHARGE syndrome (coloboma, heart defects, atresia of the choanae, growth and development delay, genital hypoplasia, and ear anomalies).

## Case presentation

A 3-year-old boy was brought to our emergency department due to a change in consciousness following a seizure attack. A physical examination revealed a short stature, low body weight, spinal dysraphism, and intellectual disability. He was admitted for acute and chronic subdural haemorrhage, pneumothorax and multiple rib fractures. The boy was claimed to have suffered head trauma at home, and his mother stated that he had longstanding poor vision. An ophthalmologist was consulted for the possibility of injury to the eyes.

**Figure 1:**
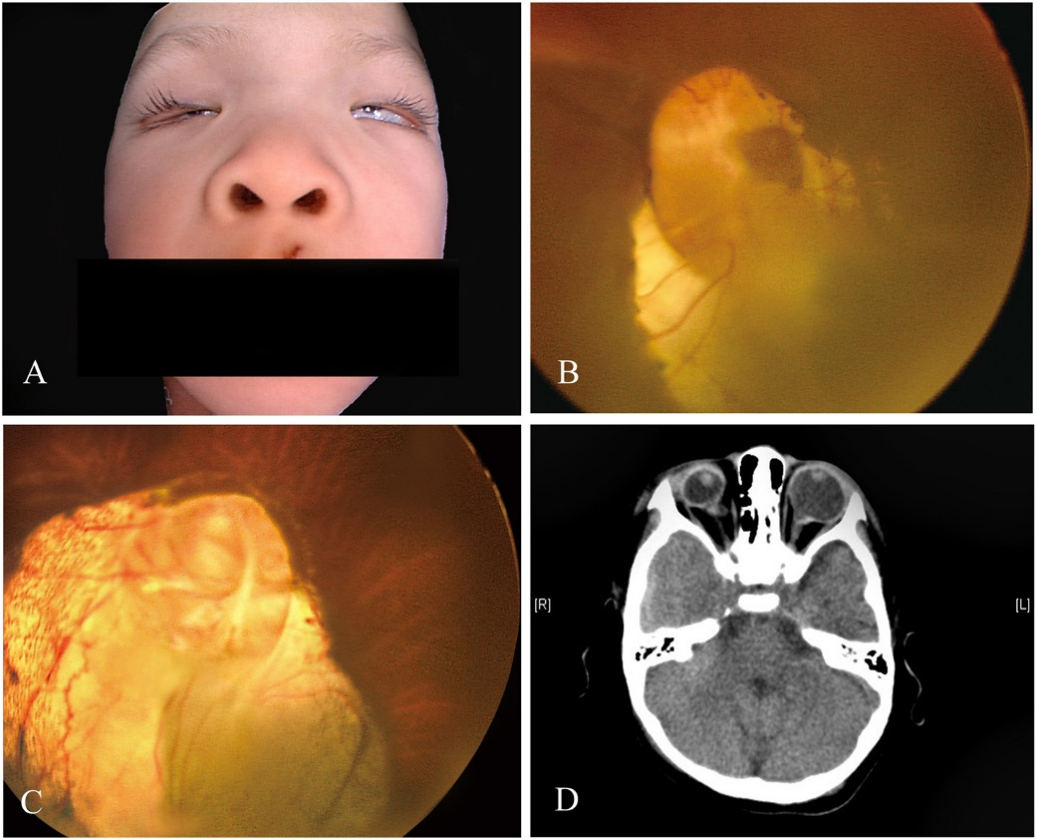
**Photographs of the 3-year-old patient with Kabuki syndrome. (A)** The external photograph shows arched eyebrows, epicanthus, ptosis, prominent eyelashes, long palpebral fissures, eversion of the lateral portion of the lower eyelids in both eyes, and depressed nasal tip. **(B)** The fundus photograph of the right eye shows coloboma of the retina, choroid and disc. **(C)** The fundus photograph of the left eye shows coloboma of the retina and choroid and an elevated optic disc without central cupping. **(D)** Computed tomography of the orbit reveals microphthalmia of the right eye and an isodense retrobulbar bulging cystic mass in both eyes. The defect is likely to be related to the colobomatous defect presented in the fundus photograph. No orbital fractures or intraorbital haemorrhages were found.

The child had peculiar facial features with a depressed nasal tip, low-set deformed ears, and micrognathia. The external ophthalmic examination revealed arched eyebrows, epicanthus, ptosis, prominent eyelashes, long palpebral fissures, and eversion of the lateral portion of the lower eyelids in both eyes. In addition, microphthalmia in the right eye was noted (Figure 1A). The child had bilateral horizontal nystagmus and esotropia with limitation of abduction of 30 to 40% in the left eye. The visual acuity was no light perception in the right eye and counting finger(s) in the left eye. An examination under anaesthesia revealed a normal cornea in the left eye but a microcornea in the right eye. The anterior chamber and lens were normal in both eyes. Iris and optic disc coloboma were discovered in the right eye (Figure 1B). In addition, retinal and choroidal coloboma in both eyes and an elevated optic disc without central cupping in the left eye were found (Figure 1C). There was no evidence of retinal or vitreous haemorrhage. Computed tomography of the orbit showed isodense retrobulbar bulging cystic masses in both eyes, which were likely related to the colobomatous defect. In addition, colobomatous microphthalmia in the right eye was discovered. No orbital fracture or intraorbital haemorrhage was found (Figure 1D). A diagnosis of shaken baby syndrome was excluded based on these findings.

A detailed review of the literature illustrates that various ophthalmic abnormalities have been associated with Kabuki syndrome. In the present case, there was neither a history of consanguinity in the parents nor peculiar faces in other family members. Further genetic study revealed that the patient had a normal 46XY chromosome pattern but an MLL2 gene mutation.

## Discussion

The prevalence of Kabuki syndrome is estimated to be 1 in 32,000 births in Japan [5]. The most common ophthalmic abnormalities in Kabuki syndrome are strabismus and ptosis, with reported incidence rates of 20.5% and 14.4%, respectively [3]. Coloboma is a less common feature that has been reported in 3.2% of the published cases [3]. Other rare abnormalities, such as nystagmus, microphthalmos, microcornea, corneal opacities, blue sclera, cataracts, nasolacrimal duct obstruction, jaw-winking type of ptosis, caruncle lipoma, corneal pannus, retinal telangiectasia, and retinal pigmentation, have been reported in Kabuki syndrome [3, 5, 9]. However, the presentation of colobomatous microphthalmia and a dysplastic disc is rare [3]. Moreover, a dysplastic disc and lack of central cupping has not been reported previously to the best of our knowledge.

Kabuki syndrome shows a phenotypic overlap with CHARGE syndrome [10–13]. Shared presentations include cleft palate, development delay, genital hypoplasia, congenital heart, and ear, eye and renal abnormalities [11–13]. Shared ophthalmic phenotypes include coloboma and staphyloma [3, 14]. CHARGE syndrome was initially considered a likely diagnosis in our patient because of the features of coloboma, growth and development delay, and ear abnormality. However, he fulfilled only two major and two minor criteria; therefore, he could not be clinically diagnosed with CHARGE syndrome. In addition, he had typical Kabuki facial presentations. The genetic survey can assist in establishing the diagnosis from a molecular level. The most common genotypic presentation in Kabuki syndrome is the MLL2 gene mutation [6–8], which contrasts to the CHD7 gene mutation found in CHARGE syndrome [11]. Our patient had an MLL2 gene mutation, which further supported the diagnosis of Kabuki syndrome.

## Conclusions

Ophthalmic abnormalities are frequently associated with Kabuki syndrome. A dysplastic elevated disc without central cupping and colobomatous microphthalmia are rare ophthalmic abnormalities in patients with Kabuki syndrome. Careful ophthalmic evaluations should be performed for each patient.

## Consent

Written informed consent was obtained from the parents of the patients for publication of this case report and any accompanying images. A copy of the written consent is available for review by the Editor-in-Chief of this journal.
